# Supplementary material for: Prevalence of type 2 diabetes mellitus and hypertension in patient’s visiting the conservative dentistry and endodontics department: a cross-sectional study in Surabaya City
Source: PeerJ. 2024 Jun 28;12:e17638. doi: 10.7717/peerj.17638 (PMC11216211; doi:10.7717/peerj.17638)
Supplement: Supplemental Information 2 [file peerj-12-17638-s002.docx]

STROBE Statement—checklist of items that should be included in reports of observational studies

|  | Item No. | Recommendation | Page  No. | Relevant text from manuscript |
| --- | --- | --- | --- | --- |
| **Title and abstract** | 1 | (*a*) Indicate the study’s design with a commonly used term in the title or the abstract | Page 1  **Lines 1 – 4** | Health trends in patients seeking endodontic treatment: a cross-sectional exploration of type 2 diabetes mellitus and hypertension prevalence in Surabaya city |
|  |  | (*b*) Provide in the abstract an informative and balanced summary of what was done and what was found | Pages 1 and 2  **Lines 29 – 53** | This descriptive cross-sectional study examined the prevalence of hypertension (HTN) and type 2 diabetes mellitus (T2DM) among patients visiting the Conservative Dentistry and Endodontics department, aiming to improve endodontic therapy delivery and ensure high-quality dental care. Prior to dental procedures, patients' blood sugar and blood pressure levels were randomly checked, with data analyzed from 614 participants (55.8% female, 44.2% male, average age 44.58 ± 12.77 years). Of these, 40.6% were referred for T2DM, 12.6% for HTN, and 24.0% for both conditions, with significant gender correlations (p < 0.05). Average blood pressure readings were 141.02 mmHg ± 56.28 mmHg (systolic), 79.83 mmHg ± 10.68 mmHg (diastolic), and 126.68 mg/dL ± 15.36 mg/dL (random blood sugar). Age correlated strongly with blood sugar and blood pressure levels (p < 0.05), with significant differences observed between referred and non-referred individuals (p < 0.05). In conclusion, patient age influences random blood sugar levels, diastolic and systolic blood pressure, urging dentists to tailor treatment plans considering these factors for optimal outcomes in managing T2DM and HTN. |
| Introduction | | | |  |
| Background/rationale | 2 | Explain the scientific background and rationale for the investigation being reported | Pages 2 and 3  **Lines 56 – 110** | This descriptive cross-sectional study focuses on the prevalence of hypertension (HTN) and type 2 diabetes mellitus (T2DM) amongst patients who visit the Conservative Dentistry and Endodontics department. Recognizing these incidence statistics is critical for improving endodontic therapy delivery and assuring high-quality dental care with positive treatment results. |
| Objectives | 3 | State specific objectives, including any prespecified hypotheses | Page 3  **Lines 100 – 110** | The main objective of this descriptive cross sectional study is to estimate the prevalence of Hypertension and type 2 diabetes mellitus among patients visiting the Department of Conservative Dentstry and Endodontics in the city of Surbaya , Indonesia |
| Methods | | | |  |
| Study design | 4 | Present key elements of study design early in the paper | Pages 4 and 5  **Lines 112 – 172** | Descriptive cross-sectional study |
| Setting | 5 | Describe the setting, locations, and relevant dates, including periods of recruitment, exposure, follow-up, and data collection | Pages 4 and 5  **Lines 112 – 172** | Patients who visited the department of Conservative Dentistry were recommended to have their blood pressure and random blood sugar levels examined before proceeding with the dental treatment. Patients who were willing for assessment were the subject of the current investigation. Patients from the city of Surabaya and surrounding areas make up our institute’s diversified patient pool |
| Participants | 6 | (*a*) *Cohort study*—Give the eligibility criteria, and the sources and methods of selection of participants. Describe methods of follow-up  *Case-control study*—Give the eligibility criteria, and the sources and methods of case ascertainment and control selection. Give the rationale for the choice of cases and controls  *Cross-sectional study*—Give the eligibility criteria, and the sources and methods of selection of participants | Page 4 and 5  **Lines 140 – 159** | Patients who visited the department of Conservative Dentistry were recommended to have their blood pressure and random blood sugar levels examined before proceeding with the dental treatment. Patients who were willing for assessment were the subject of the current investigation. Patients from the city of Surabaya and surrounding areas make up our institute’s diversified patient pool |
|  |  | (*b*) *Cohort study*—For matched studies, give matching criteria and number of exposed and unexposed  *Case-control study*—For matched studies, give matching criteria and the number of controls per case |  |  |
| Variables | 7 | Clearly define all outcomes, exposures, predictors, potential confounders, and effect modifiers. Give diagnostic criteria, if applicable | Page 4 and 5  **Lines 140 – 159** | If the average blood pressure readings still remained 130/90 mm Hg and above, the patients were advised to visit a nearby hospital for the appropriate control of blood pressure before returning for their dental treatment  If the blood sugar reading were above 126 gm/dl, the patients were asked to visit a nearly hospital for the appropriate control of blood sugar before returning for their dental treatment. |
| Data sources/ measurement | 8* | For each variable of interest, give sources of data and details of methods of assessment (measurement). Describe comparability of assessment methods if there is more than one group | Page 4 and 5  **Lines 140 – 159** | Patients were instructed to relax for 15 minutes before assessing the blood pressure. While assessing the blood pressure, patients were instructed to sit comfortably in an upright position while their blood pressure was measured using a digital sphygmomanometer (BPL Blood Pressure Monitor, Bengaluru, India), and the results were reported and categorised according to classification shown in (Table 1) (Carey & Whelton, 2018). If the blood pressure readings were normal, patients were informed to carry out their treatment. However, If the readings were higher than 130/90, the patients were instructed to unwind comfortably for 30 minutes before having their blood pressure checked again. The results were once again noted, and the average was taken into account. If the average readings still remained 130/90 mm Hg and above, the patients were advised to visit a nearby hospital for the appropriate control of blood pressure before returning for their dental treatment  For recording Blood sugar levels, the patients were made to sit in comfortable position and the readings were recorded with the help of a glucometer (Glucospark glucometer, Sensa core Medical Instrumentation, Telangana, India). If the readings were less than 126 gm/dl, the patients were informed to continue with the treatment. If the reading were above 126 gm/dl, the patients were asked to visit a nearly hospital for the appropriate control of blood sugar before returning for their dental treatment |
| Bias | 9 | Describe any efforts to address potential sources of bias | - | There was no bias in our study |
| Study size | 10 | Explain how the study size was arrived at | Page 4  **Lines 133 – 138** | Sample size was estimated using the data from previous study (Bogari & Bakalka, 2016) employing the formula [DEFF*Np(1-p)]/ [(d 2 /Z 2 1-α/2 *(N-1) + p*(1-p)], where Design effect (for cluster surveys-DEFF) is 1, hypothesized frequency of 63.7% * at 99.9%, Confidence Level with 0.1% precision(d). The total sample size estimated was 1001. However, 1100 subjects were included in the study. |

Continued on next page

| Quantitative variables | 11 | Explain how quantitative variables were handled in the analyses. If applicable, describe which groupings were chosen and why | Page 4 and 5  **Lines 140 – 159** | **Recording and classifying patient’s blood pressure levels**  **Recording and classifying patient’s plasma glucose levels** |
| --- | --- | --- | --- | --- |
| Statistical methods | 12 | (*a*) Describe all statistical methods, including those used to control for confounding | Page 5  **Lines 161 – 172** | The data was accurately entered from the departmental records in Microsoft Excel Version 13 and was sent for statistical analysis (IBM SPSS version 21). Frequency Percentage was obtained for Categorical Variable (Gender, Location, Referral) and for Continuous variable (Systolic Blood Pressure, Diastolic Blood Pressure and Random Blood Sugar Level) Mean SD was obtained. To compare Systolic Blood Pressure, Diastolic Blood Pressure and Random Blood Sugar Level between Gender and Location unpaired T test was applied. To compare between Systolic Blood Pressure, Diastolic Blood Pressure and Random Blood Sugar Level between referral of the patients ANOVA was applied. Pearson Correlation was done to observe the correlation between Age and Systolic Blood Pressure, Diastolic Blood Pressure and Random Blood Sugar Level of the patients. All the tests were applied keeping confidence interval at 95% and (p<0.05) was considered to be statistically significant |
|  |  | (*b*) Describe any methods used to examine subgroups and interactions | Page 5  **Lines 161 – 172** |  |
|  |  | (*c*) Explain how missing data were addressed |  |  |
|  |  | (*d*) *Cohort study*—If applicable, explain how loss to follow-up was addressed  *Case-control study*—If applicable, explain how matching of cases and controls was addressed  *Cross-sectional study*—If applicable, describe analytical methods taking account of sampling strategy |  |  |
|  |  | (*e*) Describe any sensitivity analyses | Page 5  **Lines 161 – 172** |  |
| Results | | | | |
| Participants | 13* | (a) Report numbers of individuals at each stage of study—eg numbers potentially eligible, examined for eligibility, confirmed eligible, included in the study, completing follow-up, and analysed | Page 5  **Lines 175 – 177** | For the current study, the minimum age of participants was 17 years and the maximum age was 90 years. The mean age of the participants was 44.58 ± 12.77  Of the 1100 individuals, 486 (44.2%) were male and 614 (55.8%) were female. There were 250 (22.7%) Normal Patients and 447 (40.6%) Type 2 Diabetic Patients that were referred to the hospital. 264 (24.0%) patients were referred to the hospital for both type 2 diabetes and hypertension whereas 139 (12.6%) patients were referred to the medical hospital for hypertension alone |
|  |  | (b) Give reasons for non-participation at each stage |  |  |
|  |  | (c) Consider use of a flow diagram |  |  |
| Descriptive data | 14* | (a) Give characteristics of study participants (eg demographic, clinical, social) and information on exposures and potential confounders | Pages 5 and 6  **Lines 179 – 214** | For the current study, the minimum age of participants was 17 years and the maximum age was 90 years. The mean age of the participants was 44.58 ± 12.77  Of the 1100 individuals, 486 (44.2%) were male and 614 (55.8%) were female. There were 250 (22.7%) Normal Patients and 447 (40.6%) Type 2 Diabetic Patients that were referred to the hospital. 264 (24.0%) patients were referred to the hospital for both type 2 diabetes and hypertension whereas 139 (12.6%) patients were referred to the medical hospital for hypertension alone |
|  |  | (b) Indicate number of participants with missing data for each variable of interest |  |  |
|  |  | (c) *Cohort study*—Summarise follow-up time (eg, average and total amount) |  |  |
| Outcome data | 15* | *Cohort study*—Report numbers of outcome events or summary measures over time |  |  |
|  |  | *Case-control study—*Report numbers in each exposure category, or summary measures of exposure |  |  |
|  |  | *Cross-sectional study—*Report numbers of outcome events or summary measures | Pages 5-6  **Lines 174 – 214** | Result section |
| Main results | 16 | (*a*) Give unadjusted estimates and, if applicable, confounder-adjusted estimates and their precision (eg, 95% confidence interval). Make clear which confounders were adjusted for and why they were included |  |  |
|  |  | (*b*) Report category boundaries when continuous variables were categorized |  |  |
|  |  | (*c*) If relevant, consider translating estimates of relative risk into absolute risk for a meaningful time period |  |  |

Continued on next page

| Other analyses | 17 | Report other analyses done—eg analyses of subgroups and interactions, and sensitivity analyses | Page 6  **Lines 195 – 214** | Not applicable |
| --- | --- | --- | --- | --- |
| Discussion | | | | |
| Key results | 18 | Summarise key results with reference to study objectives | Pages 6 – 10  **Lines 216 – 359** | **Discussion Section** |
| Limitations | 19 | Discuss limitations of the study, taking into account sources of potential bias or imprecision. Discuss both direction and magnitude of any potential bias | 9 and 10  **Line 346 – 352** | This study's single-center emphasis on patients from the Department of Conservative Dentistry in Surabaya presents reservations regarding the generalizability of the results to a larger population, among other potential shortcomings. Although useful for determining prevalence at an instance, the cross-sectional methodology is not able to adequately represent the dynamic character of type 2 diabetes and hypertension over an extended period of time. The study might not accurately reflect the socioeconomic and cultural variety found in the general community, even if it includes a range of age groups, genders, and referral patterns. |
| Interpretation | 20 | Give a cautious overall interpretation of results considering objectives, limitations, multiplicity of analyses, results from similar studies, and other relevant evidence | Pages 6 – 10  **Lines 216 – 359** | **Discussion Section** |
| Generalisability | 21 | Discuss the generalisability (external validity) of the study results | Page 10  **Lines 353 – 359** | In order to reflect a broad patient population, future research should use a longitudinal approach, do multicenter studies, and investigate the socioeconomic variables that affect the prevalence of diabetes and hypertension in dental patients. While integrating biomarkers and working with medical experts can improve accuracy, a crucial research path continues to be examining the interactions between the oral microbiota, diabetes, hypertension, and systemic health. Comprehensive patient care requires an understanding of how endodontic therapies affect the systemic health of individuals with diabetes and hypertension. |
| Other information | |  | | |
| Funding | 22 | Give the source of funding and the role of the funders for the present study and, if applicable, for the original study on which the present article is based |  | No fundings were required for this study. |

*Give information separately for cases and controls in case-control studies and, if applicable, for exposed and unexposed groups in cohort and cross-sectional studies.

**Note:** An Explanation and Elaboration article discusses each checklist item and gives methodological background and published examples of transparent reporting. The STROBE checklist is best used in conjunction with this article (freely available on the Web sites of PLoS Medicine at http://www.plosmedicine.org/, Annals of Internal Medicine at http://www.annals.org/, and Epidemiology at http://www.epidem.com/). Information on the STROBE Initiative is available at www.strobe-statement.org.
